# Supplementary material for: Bacterial and host enzymes modulate the inflammatory response produced by the peptidoglycan of the Lyme disease agent
Source: bioRxiv. 2025 Jan 8:2025.01.08.631998. Preprint. [Version 1] doi: 10.1101/2025.01.08.631998 (PMC11741416; doi:10.1101/2025.01.08.631998)
Supplement: Supplementary file 1 [file NIHPP2025.01.08.631998v1-supplement-2.pdf]

bioRxiv preprint doi: https://doi.org/10.1101/2025.01.08.631998; this version posted January 8, 2025. The copyright holder for this preprint (which was not certified by peer review) is the author/funder, who has granted bioRxiv a license to display the preprint in perpetuity. It is made available under aCC-BY 4.0 International license.

Supplemental Materials: Bacterial and host enzymes modulate the inflammatory response produced by the peptidoglycan of the Lyme disease agent

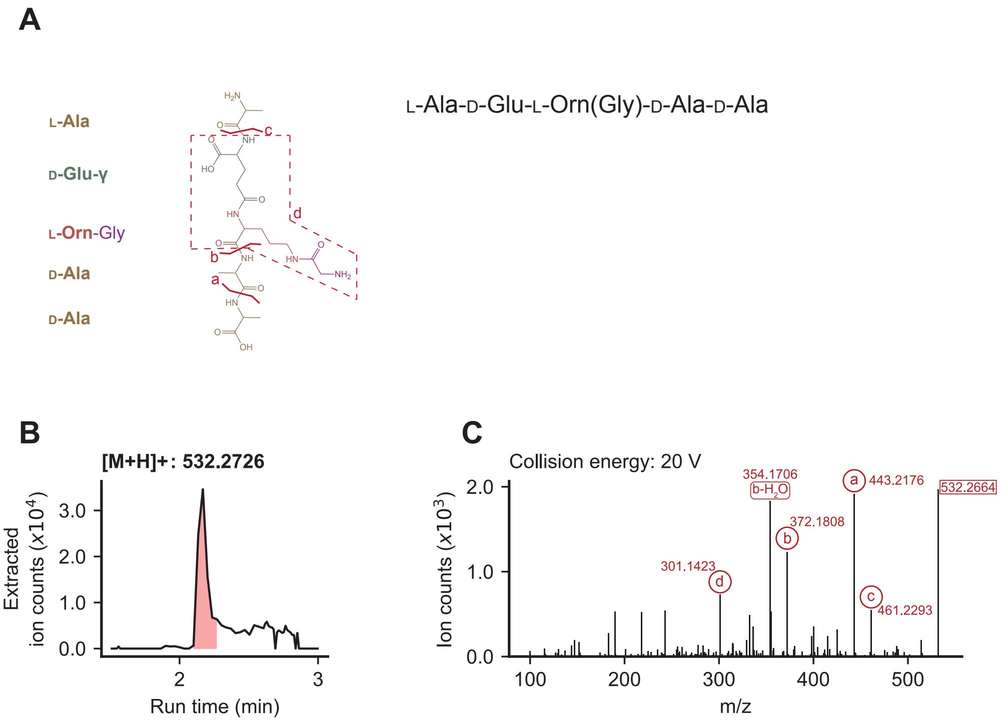

Fig. S1. Fragmentation of PG[Bb] fragment L-Ala-D-Glu-L-Orn(Gly)-D-Ala-D-Ala. **A.** Schematic outlining each amino acid in L-Ala-D-Glu-L-Orn(Gly)-D-Ala-D-Ala, along with notations of the origin of each identified fragment after MS/MS. **B.** EIC profile of the identified [M+H]+ profile. The peak of interest is shaded in red. **C.** The MS2 spectrum at the run time where the EIC peak in (B) is at maximum intensity. The collision energy used to fragment this molecule is indicated, and each identified fragment is marked with a letter corresponding to the schematic in (A).

bioRxiv preprint doi: https://doi.org/10.1101/2025.01.08.631998; this version posted January 8, 2025. The copyright holder for this preprint (which was not certified by peer review) is the author/funder, who has granted bioRxiv a license to display the preprint in perpetuity. It is made available under aCC-BY 4.0 International license.

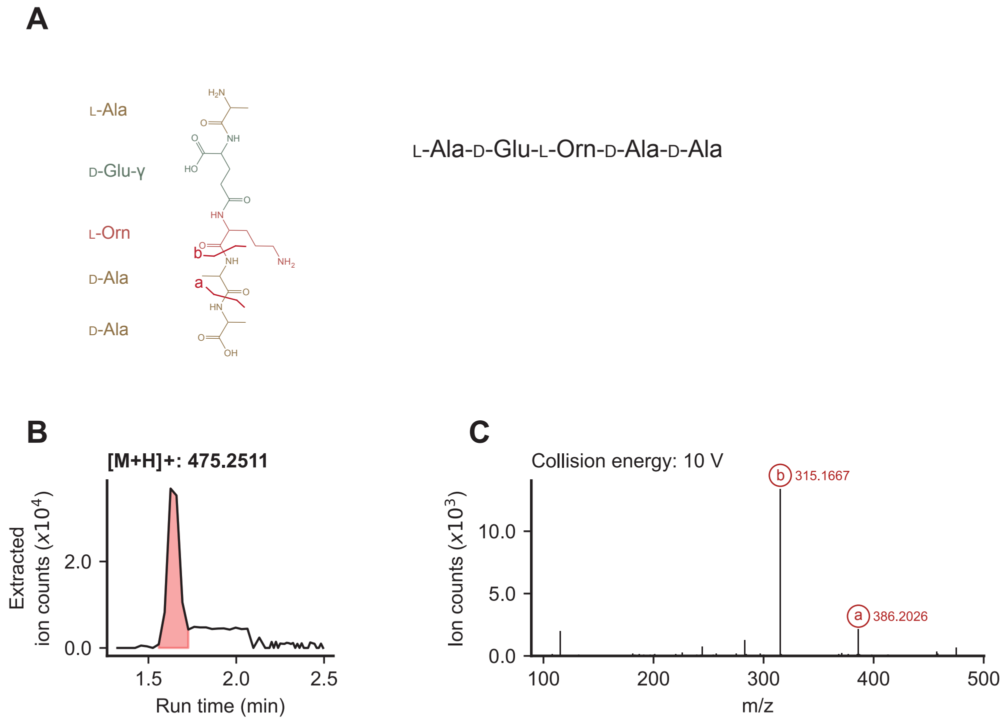

Fig. S2. Fragmentation of PG^{Bb} fragment L-Ala-D-Glu-L-Orn-D-Ala-D-Ala. **A.** Schematic outlining each amino acid in L-Ala-D-Glu-L-Orn-D-Ala-D-Ala, along with notations of the origin of each identified fragment after MS/MS. **B.** EIC profile of the identified [M+H]+ profile. The peak of interest is shaded in red. **C.** The MS2 spectrum at the run time where the EIC peak in (B) is at maximum intensity. The collision energy used to fragment this molecule is indicated, and each identified fragment is marked with a letter corresponding to the schematic in (A).

bioRxiv preprint doi: https://doi.org/10.1101/2025.01.08.631998; this version posted January 8, 2025. The copyright holder for this preprint (which was not certified by peer review) is the author/funder, who has granted bioRxiv a license to display the preprint in perpetuity. It is made available under aCC-BY 4.0 International license.

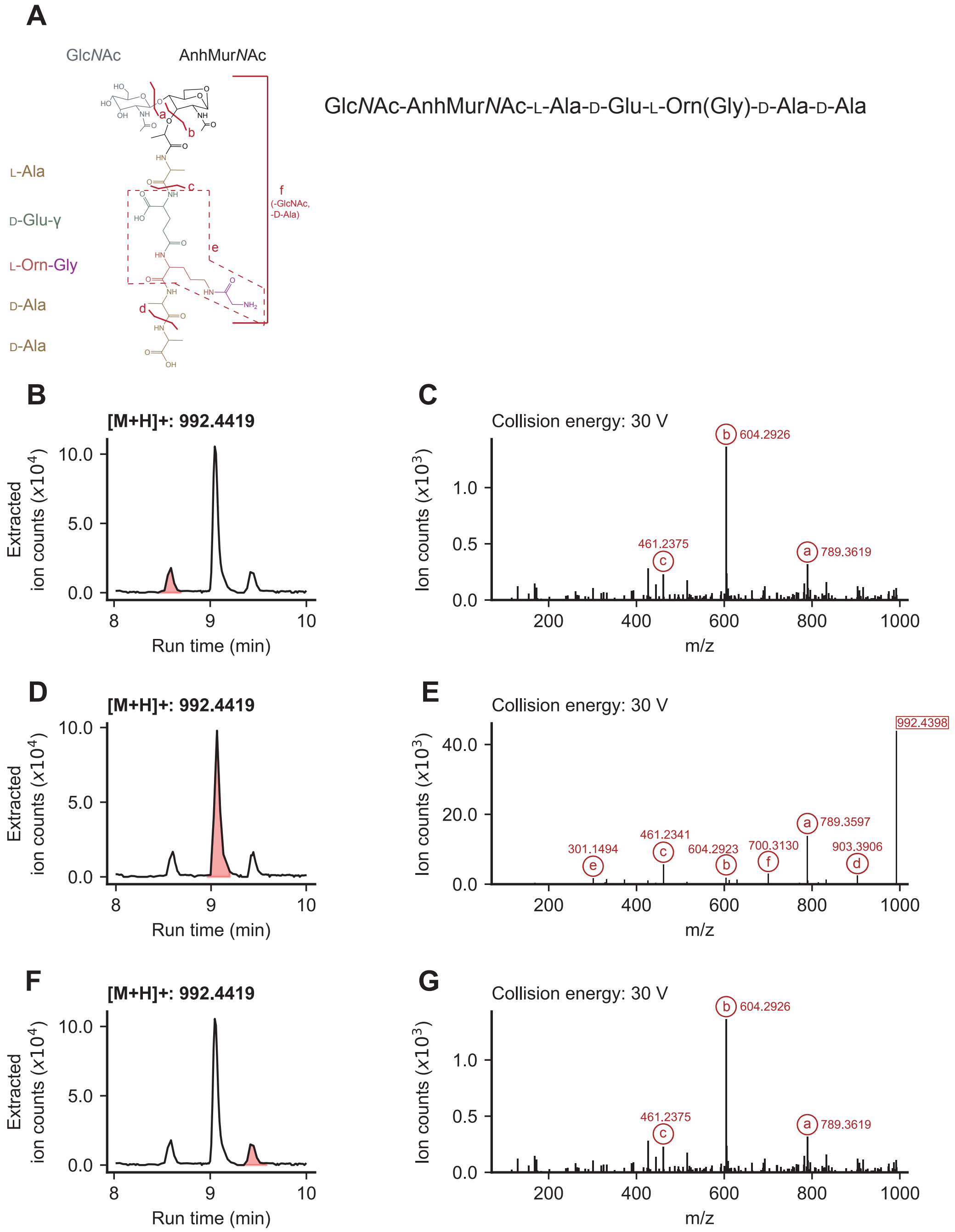

Fig. S3. Fragmentation of PG^{Bb} fragment Glc*N*Ac-AnhMur*N*Ac-L-Ala-D-Glu-L-Orn(Gly)-D-Ala-D-Ala. A. Schematic outlining the sugars and amino acids in Glc*N*Ac-AnhMur*N*Ac-L-Ala-D-Glu-L-Orn(Gly)-D-Ala-D-Ala, along with notations of the origin of each identified fragment after MS/MS. **B.** EIC profile of the identified [M+H]+ profile of peak 1, shaded in red. **C.** The MS2 spectrum at the run time where EIC peak 1 in (B) is at maximum intensity. **D.** EIC profile of the identified [M+H]+ profile of peak 2, shaded in red. **E.** The MS2 spectrum at the run time where EIC peak 2 in (D) is at maximum intensity. **F.** EIC profile of the identified [M+H]+ profile of peak 3, shaded in red. G. The MS2 spectrum at the run time where EIC peak 3 in (F) is at maximum intensity. For (C), (E), and (G), the collision energies used to fragment this molecule are noted in the titles, and each identified fragment is marked with a letter corresponding to the schematic in (A).

bioRxiv preprint doi: https://doi.org/10.1101/2025.01.08.631998; this version posted January 8, 2025. The copyright holder for this preprint (which was not certified by peer review) is the author/funder, who has granted bioRxiv a license to display the preprint in perpetuity. It is made available under aCC-BY 4.0 International license.

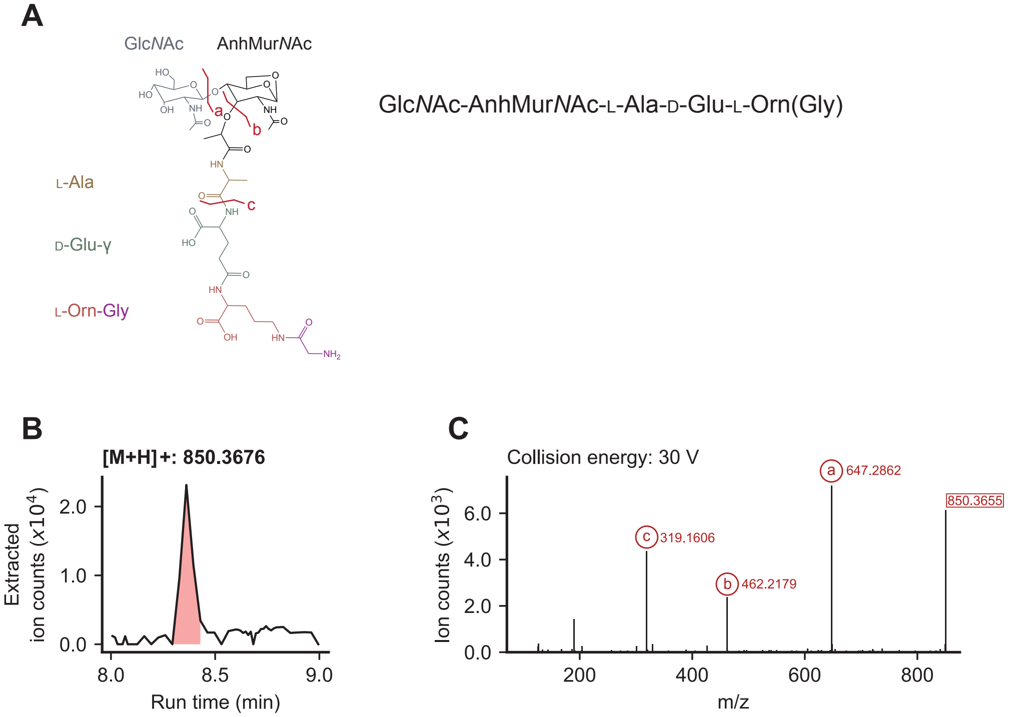

Fig. S4. Fragmentation of PG^{Bb} fragment GlcNAc-AnhMurNAc-L-Ala-D-Glu-L-Orn(Gly). A. Schematic outlining the sugars and amino acids in GlcNAc-AnhMurNAc-L-Ala-D-Glu-L-Orn(Gly), along with notations of the origin of each identified fragment after MS/MS. **B.** EIC profile of the identified [M+H]+ profile. The peak of interest is shaded in red. **C.** The MS2 spectrum at the run time where the EIC peak in (B) is at maximum intensity. The collision energy used to fragment this molecule is indicated, and each identified fragment is marked with a letter corresponding to the schematic in (A).

bioRxiv preprint doi: https://doi.org/10.1101/2025.01.08.631998; this version posted January 8, 2025. The copyright holder for this preprint (which was not certified by peer review) is the author/funder, who has granted bioRxiv a license to display the preprint in perpetuity. It is made available under aCC-BY 4.0 International license.

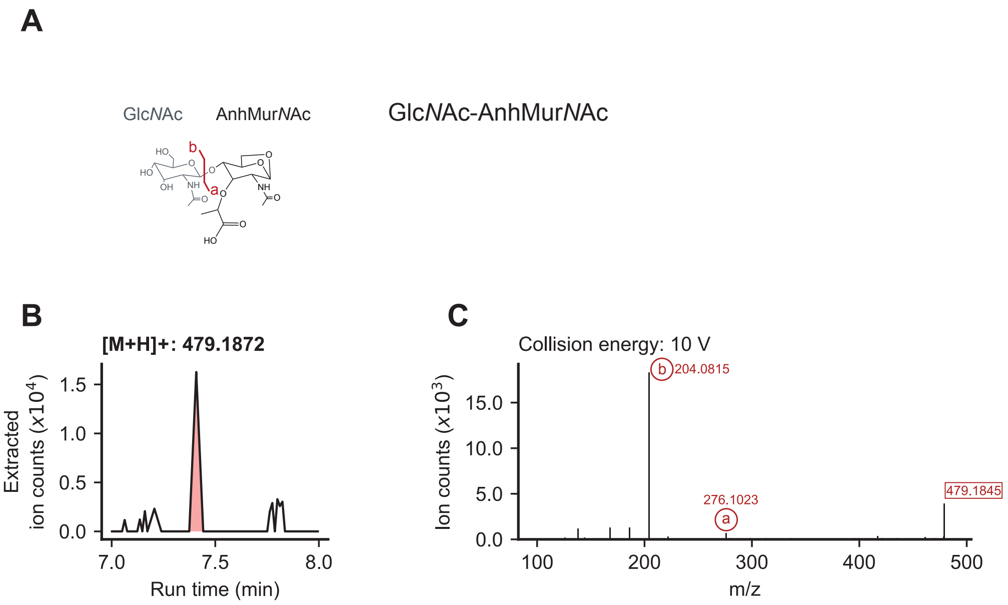

Fig. S5. Fragmentation of PG^{Bb} fragment Glc*N*Ac-AnhMur*N*Ac. A. Schematic outlining each sugar in Glc*N*Ac-AnhMur*N*Ac, along with notations of the origin of each identified fragment after MS/MS. **B.** EIC profile of the identified [M+H]+ profile. The peak of interest is shaded in red. **C.** The MS2 spectrum at the run time where the EIC peak in (B) is at maximum intensity. The collision energy used to fragment this molecule is indicated, and each identified fragment is marked with a letter corresponding to the schematic in (A).

bioRxiv preprint doi: https://doi.org/10.1101/2025.01.08.631998; this version posted January 8, 2025. The copyright holder for this preprint (which was not certified by peer review) is the author/funder, who has granted bioRxiv a license to display the preprint in perpetuity. It is made available under aCC-BY 4.0 International license.

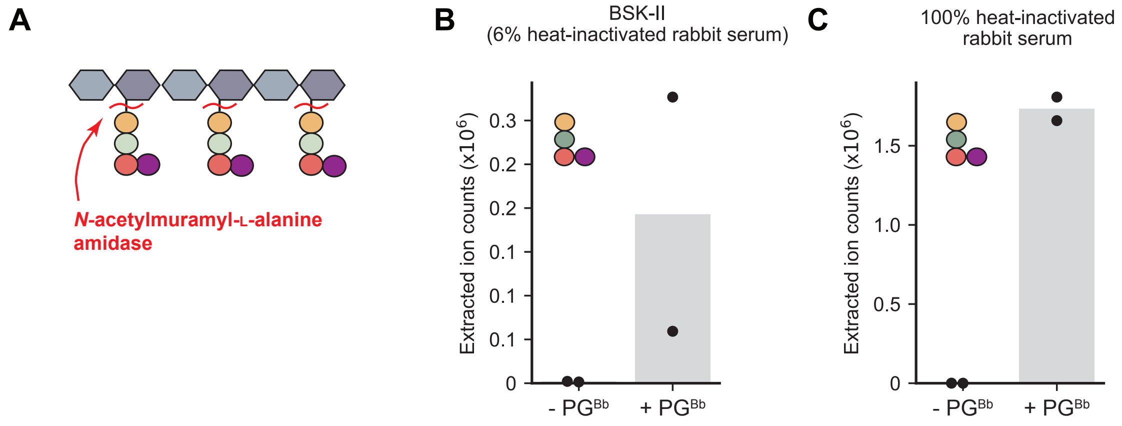

Fig. S6. *N*-acetylmuramyl-L-alanine amidase activity present in BSK-II and heat-inactivated rabbit serum. BSK-II or heat-inactivated rabbit serum was incubated at 37℃ in the presence or absence of purified PG^{Bb} sacculi for 24 h prior to LC-MS. **A.** Schematic of *N*-acetylmuramyl-L-alanine amidase activity when mixed with purified PG^{Bb}. Cut sites by a *N*-acetylmuramyl-L-alanine amidase are shown by red curvy lines. **B.** Digestion in BSK-II. **C.** Digestion in heat-inactivated rabbit serum (100%). For B-C, plots show the extracted ion count (EIC) for the L-Ala-D-Glu-L-Orn(Gly), the predominant expected digestion product of PG^{Bb} sacculi by a *N*-acetylmuramyl-L-alanine amidase. For both panels, the bar shows the mean and the dots represent the data of two biological replicates of BSK-II media and rabbit sera sourced from different lot numbers.

bioRxiv preprint doi: https://doi.org/10.1101/2025.01.08.631998; this version posted January 8, 2025. The copyright holder for this preprint (which was not certified by peer review) is the author/funder, who has granted bioRxiv a license to display the preprint in perpetuity. It is made available under aCC-BY 4.0 International license.

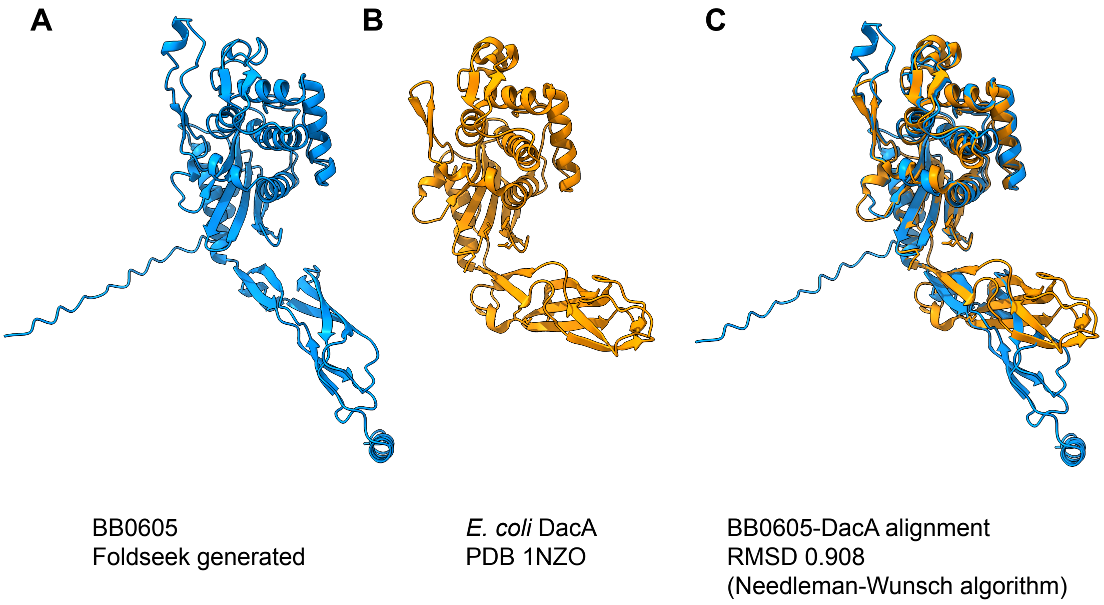

Fig. S7. Structural prediction comparison between *B. burgdorferi* BB0605 and *E. coli* DacA. A. Predicted structure of *B. burgdorferi* BB0605 using Foldseek (97) **B.** Structure of *E. coli* DacA obtained from the Protein Data Bank (PDB) (98, 99). **C.** Alignment of BB0605 and DacA, performed using ChimeraX (100). The root mean square deviation (RMSD) from alignment is presented, along with the alignment algorithm used.

bioRxiv preprint doi: https://doi.org/10.1101/2025.01.08.631998; this version posted January 8, 2025. The copyright holder for this preprint (which was not certified by peer review) is the author/funder, who has granted bioRxiv a license to display the preprint in perpetuity. It is made available under aCC-BY 4.0 International license.

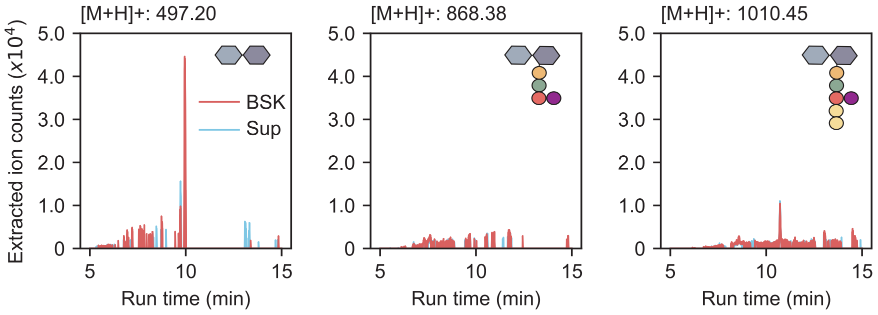

Fig. S8. Assessment of MurNAc-containing PG species in culture supernatants of *B. burgdorferi* strain K2. Plots showing the extracted ion counts of Glc*N*Ac-Mur*N*Ac, Glc*N*Ac-Mur*N*Ac-L-Ala-D-Glu-L-Orn(Gly), and Glc*N*Ac-Mur*N*Ac-L-Ala-D-Glu-L-Orn(Gly)-D-Ala-D-Ala in culture supernatants (Sup) compared to medium alone (BSK).

bioRxiv preprint doi: https://doi.org/10.1101/2025.01.08.631998; this version posted January 8, 2025. The copyright holder for this preprint (which was not certified by peer review) is the author/funder, who has granted bioRxiv a license to display the preprint in perpetuity. It is made available under aCC-BY 4.0 International license.

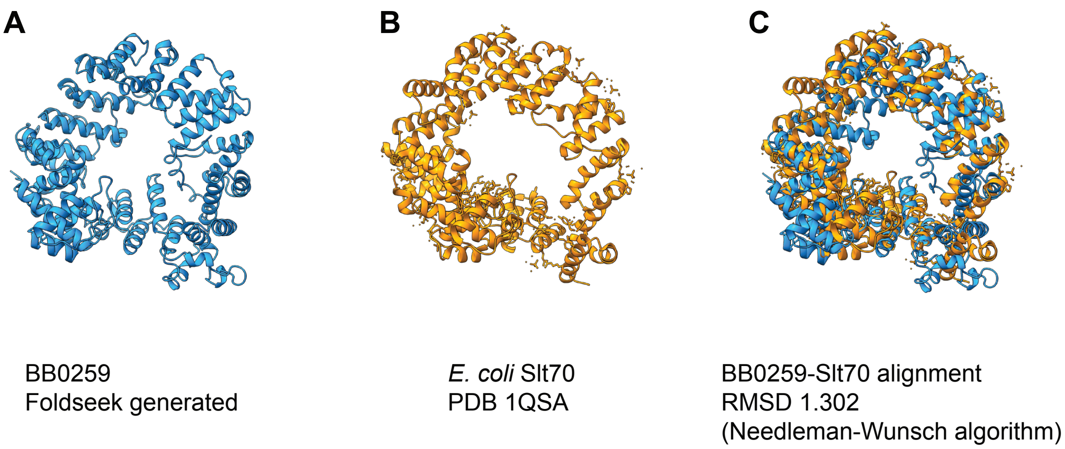

Fig. S9. Structural prediction comparison between *B. burgdorferi* BB0259 and *E. coli* Slt70. **A.** Predicted structure of BB0259 using Foldseek (97). **B.** Structure of *E. coli* Slt70 obtained from the Protein Data Bank (PDB) (98, 101). **C.** Alignment of BB0259 and Slt70 using ChimeraX (100). The root mean square deviation from alignment is presented, along with the alignment algorithm used.

bioRxiv preprint doi: https://doi.org/10.1101/2025.01.08.631998; this version posted January 8, 2025. The copyright holder for this preprint (which was not certified by peer review) is the author/funder, who has granted bioRxiv a license to display the preprint in perpetuity. It is made available under aCC-BY 4.0 International license.

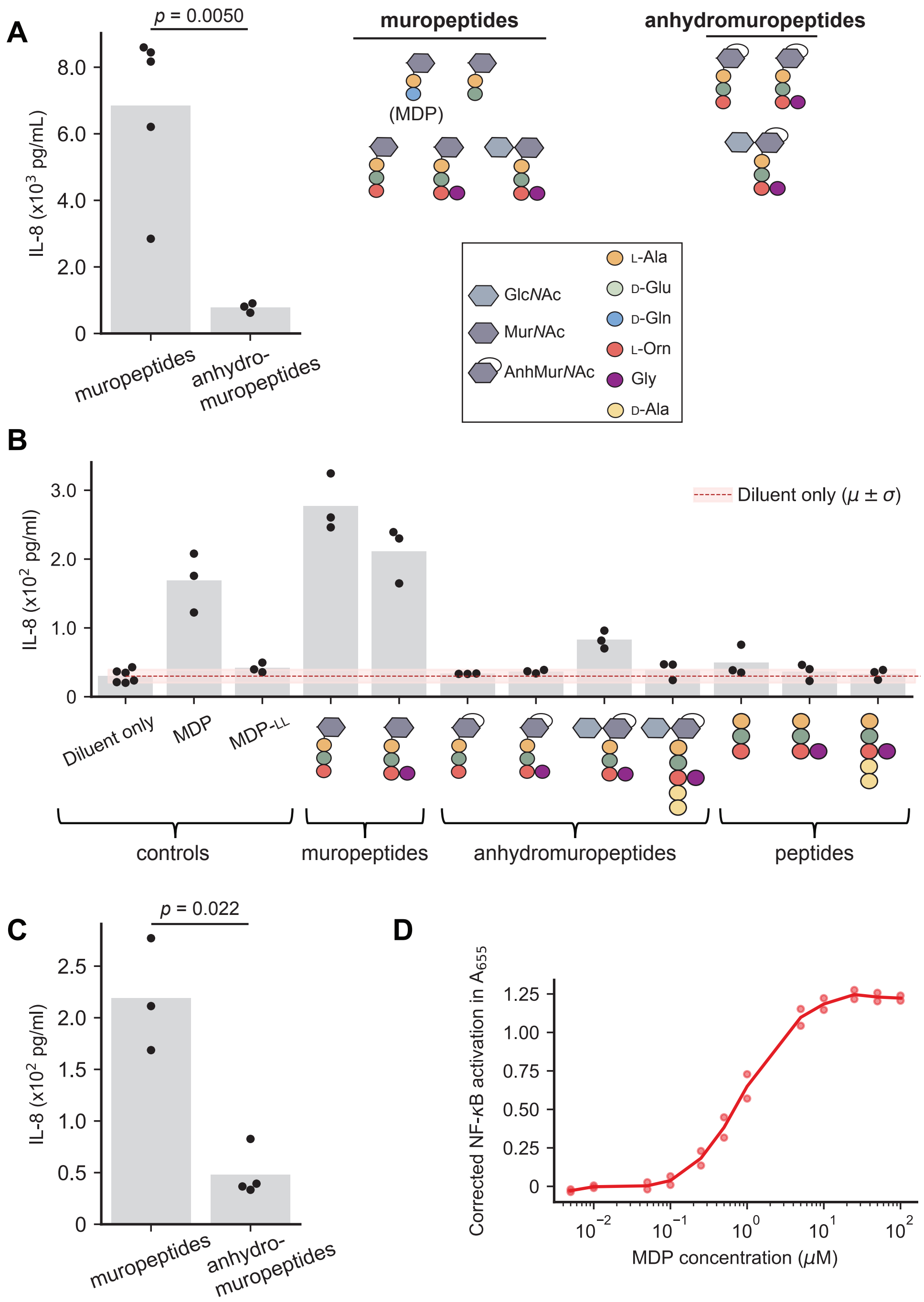

Fig. S10. Stimulation of THP-1 and hNOD2 reporter cells. **A.** Comparison between MurNAc-containing and AnhMurNAc-containing species in **Figure 5A**. The schematic of the PG^{Bb} species in each group is shown, along with a legend that defines each chemical moiety. Dots represent the means of each PG^{Bb} compound. Bar heights represent the means for each compound group. The groups were compared using a Welch's t-test to account for different standard deviations and N values. **B.** Plot showing IL-8 production in differentiated THP-1 cells in the presence of the indicated PG^{Bb} fragments. The error bars represent standard deviation of the mean, and bar height represents the mean. Dots represent data from three biological replicates. **C.** Comparison of Mur*N*Ac-containing and AnhMur*N*Ac-containing species in (B), including MDP as in (A). Dots represent the means of each PG^{Bb} compound. Bar heights represent the means for each compound group. The groups were compared using a Welch's t-test to account for different standard deviations and N values. **D.** Dose-response curve of hNOD2 reporter cells to MDP in MilliQ H_2O. The line connects the mean measurements of each concentration, and the dots represent technical replicates.

bioRxiv preprint doi: https://doi.org/10.1101/2025.01.08.631998; this version posted January 8, 2025. The copyright holder for this preprint (which was not certified by peer review) is the author/funder, who has granted bioRxiv a license to display the preprint in perpetuity. It is made available under aCC-BY 4.0 International license.

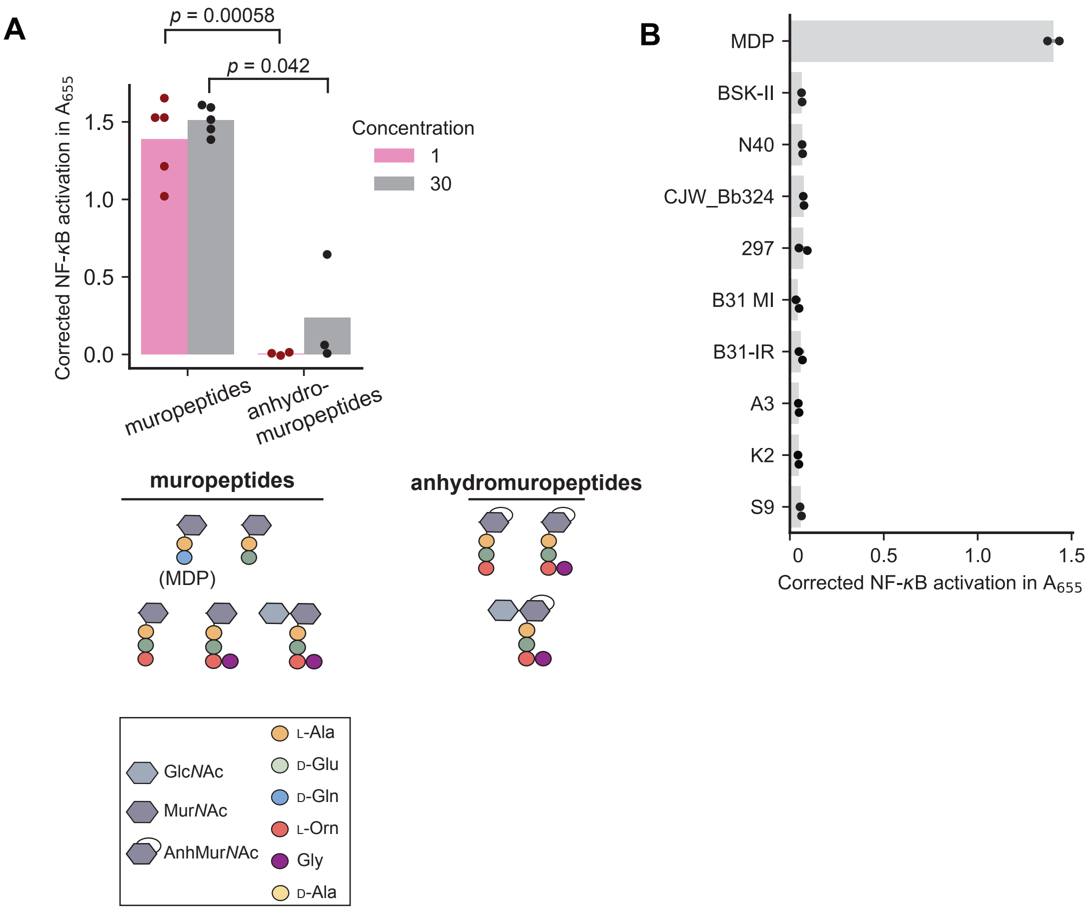

Fig. S11. Absence of detectable stimulation of hNod2 reporter cells by culture supernatants of various *B. burgdorferi* strains. **A.** NOD2 data from **Figure 5B** with PG^{Bb} grouped based on the presence of a Mur*N*Acor AnhMur*N*Ac, as shown by the schematics and the legend. Dots represent the means of each PG^{Bb} compound. Bar heights represent the means for each compound group. The lines above the plot show pairwise comparisons between each group using Welch's t-tests to account for different standard deviations and N values. The resulting p-values were adjusted using a Bonferonni correction for multiple comparisons. **B.** Plot showing the SEAP activity of hNOD2 reporter cells (used after fifth passage) following 16-h exposure to 1 μM MDP (positive control) compared to complete BSK-II medium (negative control), or supernatants of cultures in stationary phase for three days. Each dot is a technical replicate, and the height of each bar represents the mean.

bioRxiv preprint doi: https://doi.org/10.1101/2025.01.08.631998; this version posted January 8, 2025. The copyright holder for this preprint (which was not certified by peer review) is the author/funder, who has granted bioRxiv a license to display the preprint in perpetuity. It is made available under aCC-BY 4.0 International license.

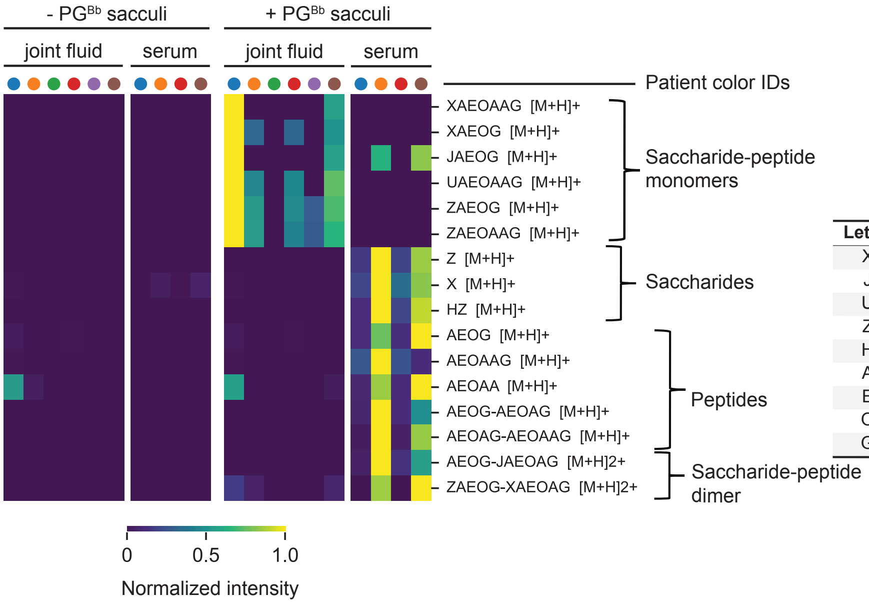

Letter	Definition
X	GlcNAc-MurNAc
J	MurNAc
U	AnhMurNAc
Z	GlcNAc-AnhMurNAc
H	GlcNAc
A	Alanine
E	Glutamate
O	Ornithine
G	Glycine

Fig. S12. Patient joint fluid and serum digestions of purified PG^{Bb} sacculi. This heatmap is similar to **Figure 6A** except that negative control are samples in which no sacculi were added. Samples were incubated with MilliQ H_2O (- PG^{Bb} sacculi) or with PG^{Bb} sacculi (+ PG^{Bb} sacculi) for 6 h, then the resulting reaction products were analyzed by LC-MS. PG^{Bb} species were detected by their predicted [M+H] value (**Supplementary Dataset 1**). All samples were derived from Lyme arthritis patients whose identities (IDs) are color-coded as in **Figure 6A**. The accompanying table contains the key to interpret the PG^{Bb} fragment species in the heatmap.

bioRxiv preprint doi: https://doi.org/10.1101/2025.01.08.631998; this version posted January 8, 2025. The copyright holder for this preprint (which was not certified by peer review) is the author/funder, who has granted bioRxiv a license to display the preprint in perpetuity. It is made available under aCC-BY 4.0 International license.

Table S1. Common PG species referenced within this manuscript. The letter key refers to th heatmap table in Figure 6A. The peptidoglycan fragment masses were generated by summing the masses of each individual subunit and substracting the mass of water with each addition.

№	PG fragment candidate	Letter Key	Calculated mass	Detected mass	Representative image
1	L-Ala-D-Glu-L-Orn(Gly)-D-Ala-D-Ala	AEOAAG	531.2652745	532.2725505 [M+H]+	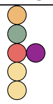
2	L-Ala-D-Glu-L-Orn-D-Ala-D-Ala	AEOAA	474.2438112	475.2510872 [M+H]+	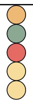
3	Glc*N*Ac-AnhMur*N*Ac-L-Ala-D-Glu-L-Orn(Gly)-D-Ala-D-Ala	ZAEOAAG	991.4345841	992.4418601 [M+H]+	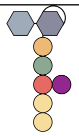
4	Glc*N*Ac-AnhMur*N*Ac-L-Ala-D-Glu-L-Orn(Gly)	ZAEOG	849.3603575	850.3676335 [M+H]+	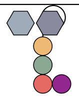
5	Glc*N*Ac-AnhMur*N*Ac	Z	478.1798743	479.1871503 [M+H]+	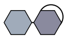
6	L-Ala-D-Glu-L-Orn	AEO	332.1695846	333.1768606 [M+H]+	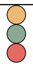
7	L-Ala-D-Glu-L-Orn(Gly)	AEOG	389.1910479	390.1983239 [M+H]+	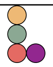
8	Glc*N*Ac-Mur*N*Ac-L-Ala-D-Glu-L-Orn(Gly)	XAEOG	867.3709225	868.3781985 [M+H]+	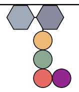
9	Glc*N*Ac-Mur*N*Ac-L-Ala-D-Glu-L-Orn(Gly)-D-Ala-D-Ala	XAEOAAG	1009.445149	1010.452425 [M+H]+	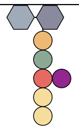
10	Glc*N*Ac-Mur*N*Ac	X	496.1904393	497.1977153 [M+H]+	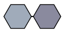
11	Mur*N*Ac-L-Ala-D-Glu	JAE	493.1907736	494.1980496 [M+H]+	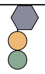
12	Mur*N*Ac-L-Ala-D-Glu-L-Orn	JAEO	607.2700869	608.2773629 [M+H]+	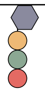
13	Mur*N*Ac-L-Ala-D-Glu-L-Orn(Gly)	JAEOG	664.2915502	665.2988262 [M+H]+	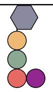
14	AnhMur*N*Ac-L-Ala-D-Glu-L-Orn	UAEO	589.2595219	590.2667979 [M+H]+	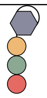
15	AnhMur*N*Ac-L-Ala-D-Glu-L-Orn(Gly)	UAEOG	646.2809852	647.2882612 [M+H]+	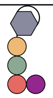
16	L-Ala-D-Glu-L-Orn[Gly—D-Ala-L-Orn(Gly)-D-Glu-L-Ala]	AEOG-AEOAG	831.4086444	832.4159204 [M+H]+ 416.7115982 [M+H]2+	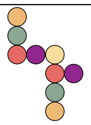
17	L-Ala-D-Glu-L-Orn[Gly—D-Ala-L-Orn(Gly)-D-Glu-L-Ala]-D-Ala-D-Ala	AEOAAG-AEOAG	973.482871	974.490147 [M+H]+ 487.7487115 [M+H]2+	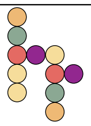

bioRxiv preprint doi: https://doi.org/10.1101/2025.01.08.631998; this version posted January 8, 2025. The copyright holder for this preprint (which was not certified by peer review) is the author/funder, who has granted bioRxiv a license to display the preprint in perpetuity. It is made available under aCC-BY 4.0 International license.

18	Mur*N*Ac-L-Ala-D-Glu-L-Orn(Gly)-D-Ala-D-Ala	JAEOAAG	806.3657768	807.3730528 [M+H]+	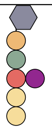
19	AnhMur*N*Ac-L-Ala	UA	346.1376153	347.1448913 [M+H]+ 174.0760837 [M+H]2+	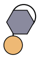
20	AnhMur*N*Ac-L-Ala-D-Glu-L-Orn(Gly)-D-Ala-D-Ala	UAEOAAG	788.3552118	789.3624878 [M+H]+	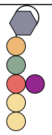
21	Glc*N*Ac-Glc*N*Ac-AnhMur*N*Ac	HZ	681.2592396	682.2665156 [M+H]+	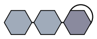
22	L-Ala-D-Glu-L-Orn[Gly–D-Ala-L-Orn(Gly)-D-Glu-L-Ala-Mur*N*Ac]	AEOG-JAEOAG	1106.509147	1107.516423 [M+H]+ 554.2618494 [M+H]2+	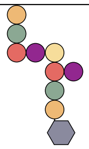
23	Glc*N*Ac-AnhMur*N*Ac-L-Ala-D-Glu-L-Orn[Gly–D-Ala-L-Orn(Gly)-D-Glu-L-Ala-Mur*N*Ac-Glc*N*Ac]	ZAEOG-XAEOAG	1769.757829	1770.765105 [M+H]+ 885.8861903 [M+H]2+	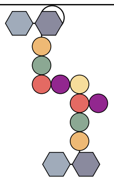

bioRxiv preprint doi: https://doi.org/10.1101/2025.01.08.631998; this version posted January 8, 2025. The copyright holder for this preprint (which was not certified by peer review) is the author/funder, who has granted bioRxiv a license to display the preprint in perpetuity. It is made available under aCC-BY 4.0 International license.

Table S2. *B. burgdorferi* strains used in this study.

Strain	Relevant genotype/description	Source
B31 MI	Infectious, mouse isolate	(18)
A3	B31 MI *cp9*$^{-}$	(102)
K2	B31-A3-68 lp25[*bbe02*::P$_{flgB}$-*aphI*] lp56^{-}	(19)
S9	B31-A3-68 lp25[*bbe02*::P$_{flgB}$-*aphI*] lp56^{-}	(19)
N40	Infectious, tick isolate	(21)
N40 D10/E9	Clonal isolate of N40	(103)
CJW_Bb324	N40 D10/E9 lp25[*bbe02*::*Bbluc-aadA*]	(104)
297	Infectious, patient isolate	(20)
5A18NP1	B31, clone 5A18NP1	(105)
T08TC493	B31, clone 5A18Np1 chr[*bb0605*::*Tn*]	(25)
B31-A	Wild type, noninfectious, high passage	(36)
Δ*bb0531*	B31-A chr[*bb0531*::P$_{flgB}$-*aphI*]	(36)
Δ*bb0259*	B31-A chr[*bb0259*::P$_{flgB}$-*aphI*]	(36)
bb0259^{com}	Bb612//pBSV2G-P$_{flgB}$-BB0259	(36)
B31 MI IR (B31 IR)	B31 MI, clonal isolate	(106)

Table S3. The LC gradient for LC-MS experiments. Buffers A and B are noted in the methods.

Time (min)	A%	B%	Flow (ml/min)
0	100	0	0.2
3	100	0	0.2
7	90	10	0.2
15	80	20	0.2
17	5	95	0.2
17.5	5	95	0.4
19.5	5	95	0.4
20.5	100	0	0.4
24	100	0	0.4
24.5	100	0	0.2
26	100	0	0.2
